# Performance of self-performed SARS-CoV-2 rapid antigen test: a systematic review and meta-analysis

**DOI:** 10.3389/fpubh.2024.1402949

**Published:** 2024-10-18

**Authors:** Peiling Cai, Junren Wang, Peng Ye, Yarong Zhang, Mengping Wang, Ronglian Guo, Hongying Zhao

**Affiliations:** ^1^Department of Anatomy and Histology, School of Preclinical Medicine, Chengdu University, Chengdu, Sichuan, China; ^2^Clinical Medical College & Affiliated Hospital of Chengdu University, Chengdu, Sichuan, China; ^3^The First Affiliated Hospital of Xi'an Jiaotong University, Xi’an, Shannxi, China; ^4^Department of Pediatrics, Zhongshan Hospital of Xiamen University, Xiamen University, Xiamen, Fujian, China

**Keywords:** COVID-19, self-test, SARS-CoV-2, rapid antigen test, diagnostic accuracy

## Abstract

**Background:**

The aim of this study was to investigate the accuracy of self-tested SARS-CoV-2 rapid antigen tests.

**Methods:**

Databases of Pubmed, Embase, and Cochrane Library were searched for original studies investigating accuracy of self-tested SARS-CoV-2 rapid antigen tests, with RT-PCR as “gold standard.”

**Results:**

Forty-five eligible studies were found after database searching and screening using pre-defined criteria. The accuracy results from 50,897 suspected COVID-19 patients were pooled, and the overall sensitivity, specificity and diagnostic odds ratio were 0.77, 1.00, and 625.95, respectively. Subgroup analysis showed higher sensitivity of rapid antigen tests in subgroups of Abbott Panbio, self-collected nasal swab samples, and use of nasopharyngeal or oropharyngeal swab and lower Ct cutoff value in RT-PCR.

**Conclusion:**

Fully self-performed SARS-CoV-2 rapid antigen tests showed overall high accuracy compared to “gold standard,” and are reliable surrogates for the standard test of COVID-19 using nasopharyngeal or oropharyngeal samples and RT-PCR.

## 1 Introduction

During the COVID-19 pandemic, the rapidly spreading of the disease has casted a significant burden on healthcare systems, including the fast-growing numbers of patients in hospitals and overwhelming need for SARS-CoV-2 testing. The gold standard testing method of SARS-CoV-2 is reverse transcription-polymerase chain reaction (RT-PCR), which requires trained professional personnel to perform. In order to ease the overwhelming COVID-19 testing burden on healthcare systems, governments of many countries worldwide recommended the use of rapid antigen tests (1). Different from RT-PCR, rapid antigen tests require minimal training, and therefore allow self-testing by suspected patients. Using a long nasal swab, suspected COVID-19 patients are allowed to self-collect a fluid sample. After being dissolved in reaction buffer, the sample is then added into a test cassette containing antibody-coated nitrocellulose membrane, and visually detectable results could be obtained in less than 30 min (2). This is much shorter than RT-PCR which usually takes a couple of hours to finish. These advantages of rapid antigen tests made them very useful during the COVID-19 pandemic, and potentially in possible future disease pandemics.

Although with many advantages, the testing accuracy of rapid antigen tests has not been fully validated. This has attracted the interest of researchers. Many rapid antigen tests have been tested for their accuracy performance compared to the gold standard (RT-PCR), including Abbott BinaxNOW assay (3), Abbott Panbio COVID-19 Ag Test (4), Access Bio CareStart COVID-19 Antigen Test (5), Boson Rapid SARS-CoV-2 antigen test card (6), Roche-SD Biosensor Rapid SARS-CoV-2 Antigen Test (7), QuickNavi-COVID19 Ag kit (8), Quidel Sofia SARS IFA antigen assay (9), FAST COVID-19 SARS-CoV-2 Antigen Rapid Test Kit (10), INDICAID COVID-19 rapid antigen test (11), and etc. A previous systemic review and meta-analysis by Xie et al. analyzed the diagnostic accuracy of rapid antigen tests for SARS-CoV-2 from data of 166,943 suspected COVID-19 patients, and reported sensitivity of 0.76 and specificity of 1.00 (12). However, the sample types for SARS-Cov-2 testing in this meta-analysis involved nasopharyngeal, nasal, and other types of samples. It is known that collection of nasopharyngeal samples requires professional personnel, and could be not finished solely by the suspected COVID-19 patients themselves. Strictly speaking, rapid antigen tests using nasopharyngeal samples lack one of the key advantages of rapid antigen tests: allow self-testing, and are therefore not so useful in disease pandemics since they cannot truly ease the testing burden of healthcare systems. In addition, Xie’s meta-analysis also included LumiraDx SARS-CoV-2 Antigen Test, which requires special equipment to read out results (13), and therefore does not allow self-testing. In this systemic review and meta-analysis, only nasal samples (allow self-testing) and rapid antigen tests with no requirement of special equipment were involved, and the aim of this study was to analyze the accuracy performance of fully self-tested SARS-CoV-2 rapid antigen tests, which could hopefully help guide the large population of suspected COVID-19 patients when they intend to use SARS-CoV-2 rapid antigen tests.

## 2 Materials and methods

### 2.1 Literature searching and selection of publication

Literature search was performed by JW and XY in April 2023. Online databases (Pubmed, Embase, and Cochrane Library) were searched using the following keywords: “COVID-19,” “rapid antigen test,” “SARS coronavirus 2 test kit,” and “nasal swab,” with alternative spelling and abbreviations included (Table 1). Articles were firstly imported to Endnote software (Thomson-Reuters) and duplicated articles were removed. The rest studies were screened using the following criteria. Inclusion criteria: all original articles investigating accuracy of self-tested SARS-CoV-2 rapid antigen tests, with RT-PCR as the “gold standard.” Exclusion criteria: (1) not a human study; (2) not testing SARS-CoV-2; (3) not a rapid antigen test; (4) not self-tested; (5) not using RT-PCR as the reference method; (6) un-interpretable data. From the resulting eligible articles, accuracy data were extracted, including true positive, true negative, false positive, and false negative. The following information was also extracted: sample collector, assessed rapid antigen test, sample collection time after symptoms onset, sample type (nasal, nasopharyngeal/oropharyngeal, or combined) for rapid antigen tests and RT-PCR, region of the study, percentage of patients with symptoms, and *Ct* values used to define positive/negative of RT-PCR results. Quality assessment of diagnostic accuracy studies 2 (QUADAS-2) was used to evaluate each eligible study (14). Search results of JW and XY were compared and discussed by the two researchers. Any disagreement between JW and XY which could not solved was then solved by PC.

**Table 1:** Search strategy.

Database	Search strategy
Pubmed	((COVID-19) AND ((rapid antigen test) OR (SARS coronavirus 2 test kit))) AND ((nasal swab) OR (nose smear))
Embase	(‘nasal swab’/exp. OR ‘nasal swab’ OR (nasal AND (‘swab’/exp. OR swab))) AND (‘rapid antigen test’/exp. OR ‘sars coronavirus 2 test kit’/exp) AND ‘covid-19 testing’/exp. AND ‘article’/it
Cochrane Library	COVID-19 in Title Abstract Keyword AND rapid antigen test in Title Abstract Keyword AND nasal swab in Title Abstract Keyword

### 2.2 Statistical analysis

From the accuracy data from each eligible study, sensitivity, specificity, positive likelihood ratio (PLR), negative likelihood ratio (NLR), diagnostic odds ratio (DOR), and area under curve (AUC) of summary receiver operating characteristic (SROC) curve were pooled using Meta-DiSc 1.4 (15). When significant heterogeneity was observed during the pooling (*I^2^* ≥ 50% and *p* ≤ 0.05), random effects model was used. Otherwise, fixed effects model was used for the pooling. If significant heterogeneity was observed, threshold analysis and meta-regression were further performed. Potential publication bias in the eligible studies was assessed by Deek’s funnel plot asymmetry test using STATA 12.0 (STATA Corp.). Results were considered statistically significant if *p* < 0.05.

## 3 Results

### 3.1 Search results

**Figure 1:**
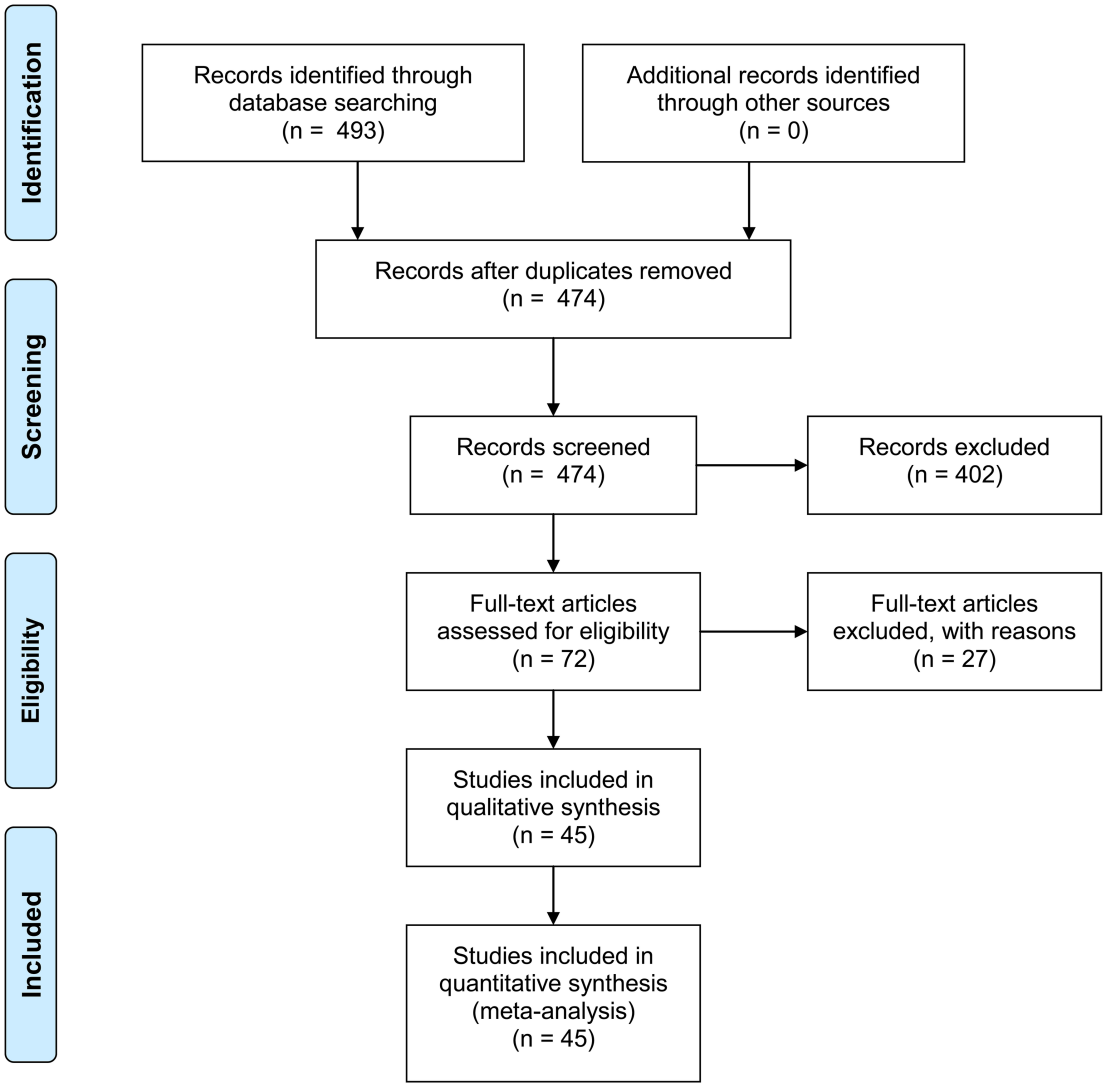
PRISMA 2009 flow diagram. From Moher et al. (55).

As shown in Figure 1, after searching the online databases, 493 publications were identified (Pubmed: 92; Embase: 398; Cochrane Library: 3), with 19 duplicated literatures. Titles and abstracts of the rest 474 publications were screened, and another 402 publications were excluded. Full-texts of the rest 72 articles were then assessed for eligibility, and another 27 articles were further excluded. Some of these 27 articles investigated tests which require special equipment to read out results (e.g., LumiraDx™ SARS-CoV-2 Antigen Test), while other articles had un-interpretable data or used samples taken from nasopharynx or throat which cannot be done by the suspected patients themselves. Accuracy data and other information were extracted from the 45 eligible studies, and meta-analysis was further performed.

### 3.2 Review of eligible publications

In the 45 eligible studies, three rapid antigen tests were intensively studied, including BinaxNOW, Panbio, and STANDARD Q. Other than these 3 tests, 23 other rapid antigen tests have also been assessed in one or two studies.

#### 3.2.1 BinaxNOW

In all, there were 11 articles investigating Abbott BinaxNOW COVID-19 antigen self test (3, 16–25). In all the studies, BinaxNOW showed high specificity (ranging from 96.51% (3) to 100% (18, 19, 25)). Sensitivity of BinaxNOW varied greatly among different studies, ranging from 20% (25) to 95.16% (3). In the two studies showing relatively low sensitivity (<50%), percentages of suspected patients with symptoms were both low (1.4% (18) and 0% (25)).

#### 3.2.2 Panbio

Seven articles reported the accuracy performance of Abbott Panbio COVID-19 Ag Rapid Test (4, 26–31). All the studies reported high specificity (from 95.45 to 100%), except one which reported specificity of 53.33%. This might be due to the relatively small sample size: 99 suspected patients, in which only 15 had negative results in RT-PCR. Sensitivity reported in these 7 articles ranged from 66 to 88.98%, in which two studies with 0% suspected patients with symptoms showed sensitivity of 66 and 88%.

#### 3.2.3 STANDARD Q

The performance of SD Biosensor STANDARD Q COVID-19 Ag test was assessed in 6 articles (32–37). Specificity of STANDARD Q ranged from 94.74 to 100%. The highest sensitivity reported for STANDARD Q was 91.18%. The lowest sensitivity was 48.48%, which was reported in a study with 0% suspected patients with symptoms.

#### 3.2.4 Other rapid antigen tests

Other than the above-mentioned rapid antigen tests, several other rapid antigen tests were also assessed in only one or two studies, including FAST COVID-19 SARS-CoV-2 Antigen Rapid Test Kit (JOYSBIO Biotechnology, China) (10), Boson Rapid SARS−CoV−2 antigen test card (Xiamen Boson, China) (6), COVID-VIRO (AAZ, France) (38), AMAZING COVID-19 Antigen Sealing Tube Test Strip (Amazing Biotech, China) (39), Zhuhai Lituo Biotechnology COVID-19 antigen detection kit (Zhuhai Lituo, China) (40), Onsite® Rapid Test (CTK Biotech, USA) (41), BIOSYNEX Antigen Self-Test COVID-19 Ag + (Biosynex Swiss, Switzerland) (42), Dräger Antigen Test SARS-CoV-2 (Dräger Safety, Germany) (43), E25Bio Rapid antigen tests (E25Bio, Inc., USA) (44), SARS-CoV-2 N rapid antigen test (self-developed) (45), SCoV-2 Ag Detect Rapid Self-Test (InBios International, Inc., USA) (46), INDICAID COVID-19 rapid antigen test (Rhino Diagnostics, USA) (11), InTec Rapid SARS-CoV − 2 Antigen Test (InTec, China) (7), Flowflex (Acon Laboratories, USA) (47), QuickNavi-COVID19 Ag kit (Denka, Japan) (8), COVID VIRO ALL IN (AAZ, France) (48), Clinitest (Siemens-Healthineers, Germany) (47), MPBio (MP Biomedicals, USA) (47), QuickNavi-Flu+COVID19 Ag kit (Denka, Japan) (49), Medomics SARS-CoV-2 antigen test (Jiangsu Medomics Medical Technology, China) (50), Roche-RDT self-testing kit (Roche Diagnostics, Switzerland) (51), CareStart COVID-19 Antigen Test (Access Bio, USA) (5, 52), and BD-RDT self-testing kit (BD Veritor, USA) (51). Similarly, specificity of these rapid antigen tests was also high, ranging from 96.35% (11) to 100% (6, 8, 38, 40, 42, 45, 49, 50). Sensitivity of these studies ranged from 49.02% (5) to 98.26% (10). Detailed sensitivity and specificity of each rapid antigen test were summarized in Table 2.

**Table 2:** Summary of sensitivity and specificity of rapid antigen tests.

Rapid antigen test	Author (year)	Sample size	Sensitivity	Specificity	% of patients with symptoms
BinaxNOW	Schrom et al. (2022) (3)	731	95.16%	96.51%	42.7 (310/731)
	Okoye et al. (2022) (16)	3,810	91.84%	99.95%	not disclosed
	Siddiqui et al. (2021) (21)	6,061	80.63%	99.78%	10.6 (642/6061)
	Ford et al. (2021) (17)	2,110	79.94%	99.89%	57.5 (1,188/2065)
	Pollock et al. (2021) (22)	2,308	77.40%	99.40%	21.8 (504/2308)
	Pollreis et al. (2021) (19)	214	67.57%	100.00%	82.7 (177/214)
	James et al. (2022) (24)	2,339	56.58%	99.86%	4.9 (115/2339)
	Sood et al. (2021) (20)	774	56.19%	98.36%	23.5 (182/774)
	Almendares et al. (2022) (23)	3,419	52.51%	99.87%	42.4 (1,451/3419)
	Surasi et al. (2021) (18)	769	43.31%	100.00%	1.4 (11/769)
	Tinker et al. (2021) (25)	1,540	20.00%	100.00%	0 (1,540/1540)
Panbio	Galliez et al. (2022) (26)	192	88.98%	100.00%	100 (192/192)
	Stokes et al. (2022) (27)	99	88.10%	53.33%	100 (99/99)
	Patriquin et al. (2022) (28)	197	88.00%	95.45%	0 (0/197)
	Klein et al. (2021) (29)	290	84.44%	99.18%	45.9 (133/290)
	Alqahtani et al. (2021) (4)	4,183	82.13%	99.13%	100 (4,183/4183)
	Sicilia et al. (2022) (30)	243	81.63%	100.00%	51 (124/243)
	Goodall et al. (2022) (31)	1,345	66.00%	100.00%	0 (1,345/1345)
STANDARD Q	Nikolai et al. (2021) (32)	96	91.18%	98.39%	93.8 (90/96)
	Sazed et al. (2022) (33)	221	86.58%	98.61%	100 (221/221)
	Lindner et al. (2021) (34)	144	82.50%	100.00%	100 (144/144)
	Begum et al. (2022) (7)	214	78.00%	94.74%	100 (214/214)
	Lee et al. (2022) (35)	175	77.46%	100.00%	not disclosed
	Sania et al. (2022) (36)	1,223	68.04%	97.75%	100 (1,223/1223)
	Jakobsen et al. (2022) (37)	7,074	48.48%	100.00%	0 (0/7074)
FAST	Polvere et al. (2022) (10)	501	98.26%	99.22%	3.2 (16/501)
Boson	Leventopoulos et al. (2022) (6)	833	98.18%	100.00%	not disclosed
COVID-VIRO	Cassuto et al. (2021) (38)	234	96.88%	100.00%	100 (234/234)
AMAZING	Medoro et al. (2022) (39)	584	92.50%	98.06%	31.3 (183/584)
Zhuhai Lituo	Terpos et al. (2021) (40)	359	91.74%	100.00%	100
Onsite	Sazed et al. (2021) (41)	380	90.98%	99.19%	100 (380/380)
BIOSYNEX	Tonen-Wolyec et al. (2021) (42)	106	90.91%	100.00%	28.3 (30/106)
Dräger	Osmanodja et al. (2021) (43)	379	88.57%	99.68%	72.0 (273/379)
E25Bio	Salcedo et al. (2022) (44)	173	86.44%	99.12%	35.8 (62/173)
SARS-CoV-2 N	Salcedo et al. (2022) (45)	23	84.62%	100.00%	not disclosed
InBios	Drain et al. (2022) (46)	797	84.44%	99.84%	100 (797/797)
INDICAID	Chiu et al. (2021) (11)	349	82.67%	96.35%	100 (349/349)
InTec	Begum et al. (2022) (7)	214	80.00%	97.37%	100 (214/214)
Flowflex	Schuit et al. (2022) (47)	620	78.97%	97.16%	100 (6,497/6497)
QuickNavi-COVID19	Takeuchi et al. (2021) (8)	862	72.55%	100.00%	91.6 (790/862)
COVID-VIRO ALL IN	Cohen et al. (2022) (48)	267	70.37%	97.85%	92.6 (774/836)
Clinitest	Schuit et al. (2022) (47)	726	70.16%	99.33%	100 (6,497/6497)
MPBio	Schuit et al. (2022) (47)	820	69.87%	98.82%	100 (6,497/6497)
QuickNavi-Flu + COVID19	Takeuchi et al. (2022) (49)	862	67.81%	100.00%	52.3 (451/862)
Medomics	Wölfl-Duchek et al. (2022) (50)	85	63.04%	100.00%	52.9 (46/87)
Roche-RDT	Stohr et al. (2022) (51)	1,583	61.46%	99.71%	69.2 (2,216/3201)
CareStart	Pollock et al. (2021) (52)	1,498	57.69%	98.34%	16.1 (241/1498)
BD-RDT	Stohr et al. (2022) (51)	1,556	49.14%	99.86%	69.2 (2,216/3201)
CareStart	Suliman et al. (2022) (5)	631	49.02%	99.48%	13.8 (87/631)

### 3.3 Quality assessment of eligible studies

QUADAS-2 was used to assess the quality of the 45 eligible studies (Table 3). In the assessment of risk of bias, high risk was observed in 3 studies (2 in patient selection, 1 in both index test and reference standard). Percentage of low risk ranged from 2% (*n* = 1, index test) to 78% (*n* = 35, flow and timing). In the assessment of application concerns, high risk was observed in 3 studies (2 in patient selection, and 2 in index test), and percentage of low risk ranged from 93% (*n* = 42, index test) to 100% (*n* = 45, reference standard).

**Table 3:** QUADAS-2 assessment of eligible studies.

Author (year)	Risk of bias			Applicability concerns			
	Patient selection	Index test	Reference standard	Flow and timing	Patient selection	Index test	Reference standard
Takeuchi et al. (2022) (49)	Unclear	Unclear	Unclear	Low	High	Low	Low
Schrom et al. (2022) (3)	Low	Unclear	Unclear	Low	Low	Low	Low
Alqahtani et al. (2022) (4)	High	Unclear	Unclear	Low	Low	Low	Low
Suliman et al. (2022) (5)	Low	Unclear	Unclear	Low	Low	Low	Low
Leventopoulos et al. (2022) (6)	Low	Unclear	Unclear	Low	Low	Low	Low
Chiu et al. (2021) (11)	Low	Unclear	Low	Low	Low	Low	Low
Galliez et al. (2022) (26)	Low	Unclear	Unclear	Low	Low	Low	Low
Tonen-Wolyec et al. (2021) (42)	Unclear	Unclear	Unclear	Low	Low	Low	Low
Begum et al. (2021) (7)	Unclear	Unclear	Unclear	Low	Low	Low	Low
Stokes et al. (2022) (27)	Unclear	Unclear	Unclear	Unclear	Low	Unclear	Low
Sania et al. (2022) (36)	Low	Unclear	Unclear	Low	Low	Low	Low
Sicilia et al. (2022) (30)	Unclear	Unclear	Unclear	Low	Low	Low	Low
Wölfl-Duchek et al. (2022) (50)	Low	Low	Low	Unclear	Low	Low	Low
Drain et al. (2022) (46)	Low	Unclear	Unclear	Unclear	Low	Low	Low
Jakobsen et al. (2021) (37)	Low	Unclear	Unclear	Unclear	Low	Low	Low
Nikolai et al. (2021) (32)	Unclear	High	High	Unclear	Low	Low	Low
Medoro et al. (2022) (39)	Low	Unclear	Unclear	Low	Low	Low	Low
Terpos et al. (2021) (40)	High	Unclear	Unclear	Low	Low	Low	Low
Sazed et al. (2021) (41)	Unclear	Unclear	Unclear	Low	Low	Low	Low
Patriquin et al. (2022) (28)	Unclear	Unclear	Unclear	Low	Low	Low	Low
Okoye et al. (2022) (16)	Unclear	Unclear	Unclear	Low	Low	Low	Low
Schuit et al. (2022) (47)	Unclear	Unclear	Low	Unclear	Low	Low	Low
Cassuto et al. (2021) (38)	Unclear	Unclear	Unclear	Low	Low	Low	Low
Pollock et al. (2021) (52)	Low	Unclear	Low	Low	Low	Low	Low
Stohr et al. (2022) (51)	Low	Unclear	Low	Unclear	Low	Low	Low
Osmanodja et al. (2021) (43)	Unclear	Unclear	Low	Low	Low	Low	Low
Ford et al. (2021) (17)	Low	Unclear	Unclear	Low	Low	Low	Low
Salcedo et al. (2022) (44)	Unclear	Unclear	Unclear	Low	Low	Low	Low
Takeuchi et al. (2021) (8)	Unclear	Unclear	Unclear	Unclear	Low	Low	Low
Surasi et al. (2021) (18)	Unclear	Unclear	Unclear	Low	Low	Low	Low
Polvere et al. (2022) (10)	Unclear	Unclear	Unclear	Unclear	Low	Low	Low
Pollreis et al. (2021) (19)	Unclear	Unclear	Unclear	Low	Low	Low	Low
Sood et al. (2021) (20)	Unclear	Unclear	Unclear	Unclear	Low	Low	Low
Siddiqui et al. (2021) (21)	Low	Unclear	Unclear	Low	Low	Low	Low
Pollock et al. (2021) (22)	Unclear	Unclear	Unclear	Low	Low	Low	Low
Almendares et al. (2022) (23)	Low	Unclear	Unclear	Low	High	High	Low
James et al. (2021) (24)	Low	Unclear	Unclear	Low	Low	Low	Low
Tinker et al. (2021) (25)	Unclear	Unclear	Unclear	Low	Low	Low	Low
Sazed et al. (2022) (33)	Unclear	Unclear	Low	Low	Low	Low	Low
Goodall et al. (2022) (31)	Unclear	Unclear	Low	Low	Low	Low	Low
Lindner et al. (2021) (34)	Unclear	Unclear	Unclear	Low	Low	Low	Low
Klein et al. (2021) (29)	Unclear	Unclear	Unclear	Low	Low	Low	Low
Cohen et al. (2022) (48)	Unclear	Unclear	Unclear	Low	Low	Low	Low
Lee et al. (2022) (35)	Unclear	Unclear	Unclear	Low	Low	High	Low
Salcedo et al. (2022) (45)	Unclear	Unclear	Unclear	Low	Low	Low	Low

Low, low risk; Unclear, unclear risk; High, high risk.

### 3.4 Meta-analysis

**Figure 2:**
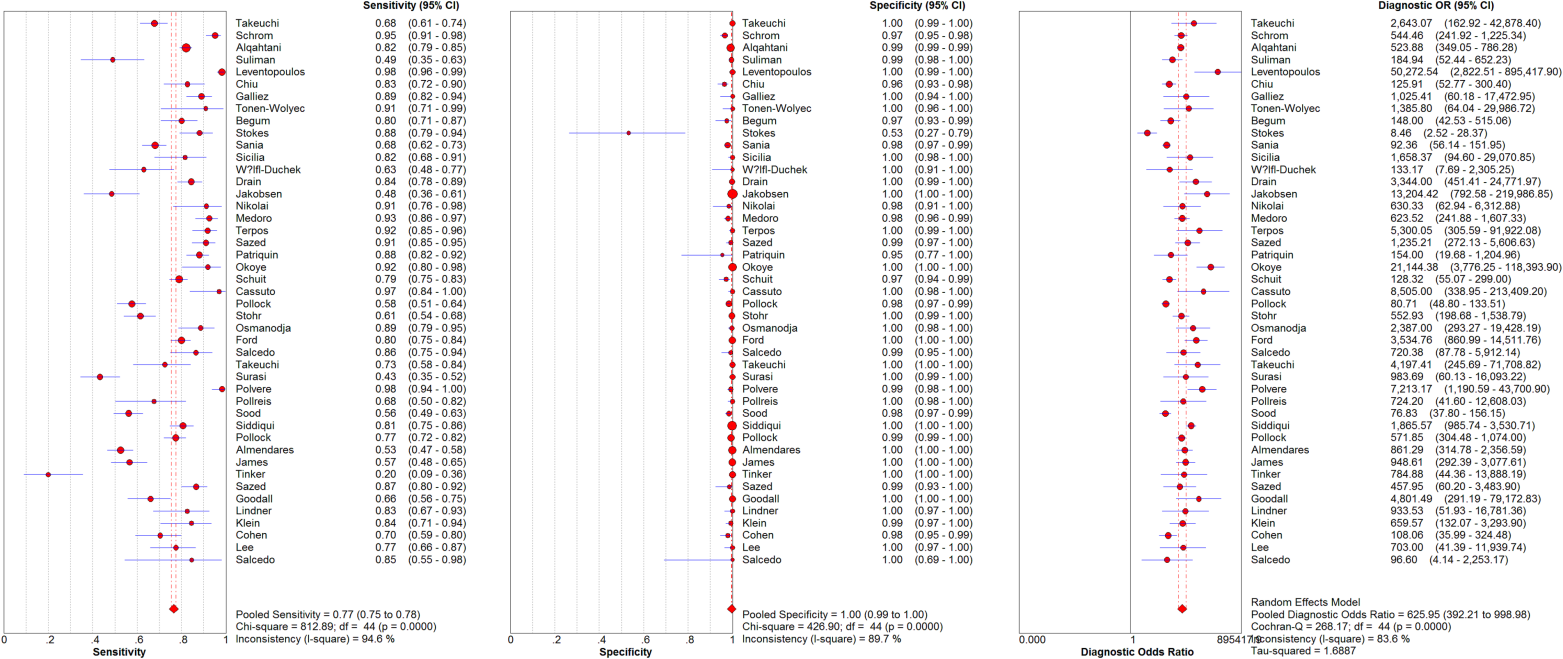
Pooled sensitivity, specificity, and DOR of eligible studies.

From the 45 eligible studies, we pooled the paired SARS-Cov-2 testing results of rapid antigen tests and RT-PCR from 50,897 suspected COVID-19 patients (see Figure 2 and Table 4). The pooled sensitivity, specificity, and DOR were 0.77 [95% confidence interval (CI): 0.75–0.78], 1.00 (95%CI: 0.99–1.00), and 625.95 (95%CI: 392.21–998.98). The AUC of SROC curve was 0.9746.

**Table 4:** Meta-analysis results.

	No. of studies	Sensitivity	Specificity	PLR	NLR	DOR	AUC of SROC curve
Overall	45	0.77 (0.75–0.78)	1.00 (0.99–1.00)	117.55 (73.68–187.54)	0.21 (0.17–0.25)	625.95 (392.21–998.98)	0.9746
Sample collector							
Self-collected	15	0.80 (0.78–0.82)	1.00 (0.99–1.00)	134.07 (59.77–300.70)	0.19 (0.12–0.29)	735.02 (314.13–1719.83)	0.9782
Healthcare professionals	20	0.73 (0.71–0.74)	1.00 (0.99–1.00)	118.47 (59.09–237.49)	0.24 (0.19–0.31)	596.90 (296.73–1200.74)	0.9751
Assessed rapid antigen test							
BinaxNOW	11	0.69 (0.67–0.71)	1.00 (1.00–1.00)	240.36 (97.73–591.18)	0.29 (0.20–0.42)	969.00 (401.75–2337.20)	0.9887
Panbio	7	0.83 (0.81–0.85)	0.99 (0.99–0.99)	68.94 (9.29–511.58)	0.18 (0.13–0.25)	367.70 (74.05–1825.87)	0.9333
STANDARD Q	6	0.73 (0.70–0.77)	1.00 (1.00–1.00)	122.18 (29.22–510.80)	0.24 (0.15–0.36)	608.98 (126.05–2942.10)	0.9520
Other tests	21	0.80 (0.78–0.81)	0.99 (0.99–0.99)	86.79 (51.89–145.18)	0.17 (0.13–0.23)	623.93 (307.20–1267.19)	0.9868
Region of study							
America	22	0.72 (0.70–0.73)	1.00 (1.00–1.00)	121.62 (54.94–269.24)	0.25 (0.20–0.33)	522.27 (250.21–1090.14)	0.9601
Europe	15	0.83 (0.81–0.85)	1.00 (1.00–1.00)	115.93 (61.34–219.10)	0.14 (0.09–0.22)	1041.31 (440.49–2461.62)	0.9905
Asia	8	0.78 (0.76–0.80)	0.99 (0.99–0.99)	81.11 (38.33–171.62)	0.21 (0.16–0.28)	406.87 (176.60–1202.77)	0.9451
Percentage of patients with symptoms							
<50%	18	0.70 (0.68–0.71)	1.00 (1.00–1.00)	127.48 (70.39–230.85)	0.26 (0.20–0.34)	639.76 (318.60–1284.67)	0.9865
≥50%	23	0.79 (0.78–0.81)	0.99 (0.99–0.99)	94.41 (44.85–198.74)	0.20 (0.17–0.24)	473.94 (247.44–907.77)	0.9327
Sample type for RT-PCR							
NP/OP swab	25	0.80 (0.79–0.82)	1.00 (1.00–1.00)	132.37 (58.73–298.32)	0.16 (0.12–0.22)	821.93 (430.13–1570.63)	0.9794
Nasal swab	16	0.73 (0.71–0.75)	1.00 (1.00–1.00)	169.78 (80.78–356.85)	0.26 (0.18–0.36)	770.74 (342.81–1732.85)	0.9788
Cutoff for Ct value							
37	5	0.78 (0.75–0.81)	1.00 (1.00–1.00)	467.11 (180.61–1208.08)	0.16 (0.07–0.39)	2593.82 (877.03–7671.22)	0.9983
40	6	0.72 (0.69–0.75)	0.99 (0.99–1.00)	78.97 (42.94–145.23)	0.24 (0.12–0.49)	470.52 (150.60–1470.02)	0.9945
Collection time after symptoms onset							
<7 days	10	0.93 (0.91–0.94)	0.99 (0.99–0.99)	94.24 (13.32–666.99)	0.09 (0.06–0.15)	969.30 (171.10–5491.34)	0.9582

PLR, positive likelihood ratio, NLR = negative likelihood ratio; DOR, diagnostic odds ratio; AUC, area under curve; SROC, summary receiver operating characteristic; RT-PCR, reverse transcription-polymerase chain reaction; NP, nasopharyngeal; OP, oropharyngeal.

During the pooling, significant heterogeneity was observed (see Figure 2). Possible sources of the heterogeneity were then investigated. Diagnostic threshold analysis indicated no significant threshold effect (Spearman correlation coefficient: 0.279, *p* = 0.063). Further meta-regression showed that inter-study heterogeneity was not associated with collector of samples (*p* = 0.7627), percentage of patients with symptoms (*p* = 0.0797), type of rapid antigen kits (*p* = 0.8037), sample type for RT-PCR (*p* = 0.7907), Ct values used to define positive/negative of RT-PCR results (*p* = 0.7744), region of studies (*p* = 0.1831), or sample collection time after symptoms onset (*p* = 0.2550). Sample type for rapid antigen tests was not included in the analysis because all the studies used nasal samples for rapid antigen tests.

Subgroup analysis were further performed based on sample collector, assessed rapid antigen test, region of study, percentage of patients with symptoms, and sample type for RT-PCR. Since the pooled specificity was quite high (1.00, 95%CI: 0.99–1.00), we focused more on the pooled sensitivity and DOR of the rapid antigen tests. Although all the rapid antigen tests involved in this systemic review could be self-performed, in some of the studies, sample collection were performed by healthcare professionals, while in other studies, samples were self-collected. After pooling the accuracy results of the two subgroups, as shown in Table 4, in the 15 studies which used self-collected samples for rapid antigen tests, the pooled sensitivity and DOR (0.80 and 735.02) were higher than the 20 studies using samples collected by healthcare professionals (0.73 and 596.90). This is an interesting result because procedures performed by healthcare professionals are normally considered to be more accurate than non-professionals. In the assessed rapid antigen tests, Abbott BinaxNOW showed the lowest pooled sensitivity (0.69) but the highest DOR (969.00). Panbio, another rapid antigen test from Abbott, showed the highest pooled sensitivity (0.83) but the lowest DOR (367.70). Regarding the region of study, studies from Europe showed the highest sensitivity (0.83) and DOR (1041.31), compared to studies from America or Asia. Studies with high percentage (≥ 50%) of patients with symptoms showed higher pooled sensitivity (0.79) but lower pooled DOR (473.94) than studies with low percentage (<50%) of patients with symptoms. For the sample type for RT-PCR, studies using nasopharyngeal or oropharyngeal samples showed higher pooled sensitivity (0.80) and DOR (821.93) compared to studies using nasal samples. This is also an interesting result because the accuracy of tests is usually considered to be higher when the same sample type is used (e.g., nasal samples used for both rapid antigen tests and RT-PCR). Since only two studies used combined nasal and nasopharyngeal/oropharyngeal samples, they were not included in the subgroup analysis.

**Figure 3:**
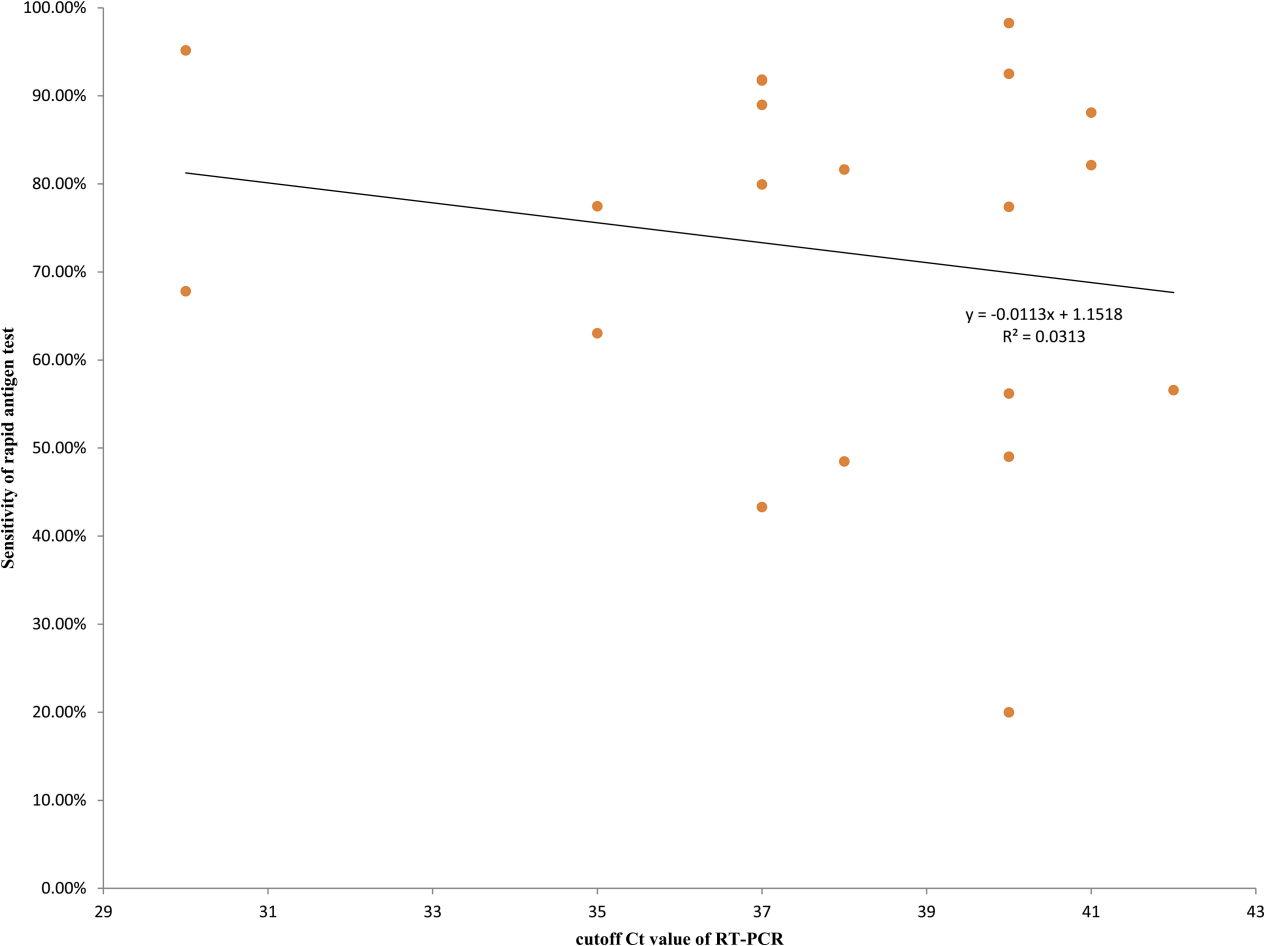
Relationship between cutoff Ct value of RT-PCR and sensitivity of rapid antigen test.

Majority of the studies (25 in 45) did not report the cutoff for Ct value. The rest studies used cutoff values of 30, 35, 37, 38, 40, 41, and 42. A study by Terpos (40) reported accuracy results when different cutoff for Ct values (25, 33, 37, and), and the results for 37 cutoff value was used because this cutoff value was more commonly used in the studies involved in this meta-analysis. When 37 was used as cutoff for Ct value, the results showed higher sensitivity (0.78) and DOR (2593.82), compared to 40 as the cutoff value (0.72 and 470.52, respectively). The rest cutoff values (30, 35, 38, 41, 42) were not included in the subgroup analysis due to limited numbers of studies using these cutoff values. Instead, we analyzed the relationship between cutoff Ct value of RT-PCR and sensitivity of rapid antigen test using a scatter plot. As shown in Figure 3, the sensitivity of rapid antigen test showed an overall decreasing trend alongwith the increase in the cutoff values of RT-PCR.

For the collection time, studies using samples collected from suspected patients within 7 days after symptoms onset showed surprisingly high sensitivity (0.93). Studies using other collection time (<5 days, <11 days, or < 14 days) were also not included in the subgroup analysis due to limited numbers of studies in these subgroups. Several studies did not report the range of collection time after symptoms onset or only reported mean and standard deviation of collection time. These studies were not included in the subgroup analysis due to the difficulty in comparing these mean ± standard deviation data with the time range.

**Figure 4:**
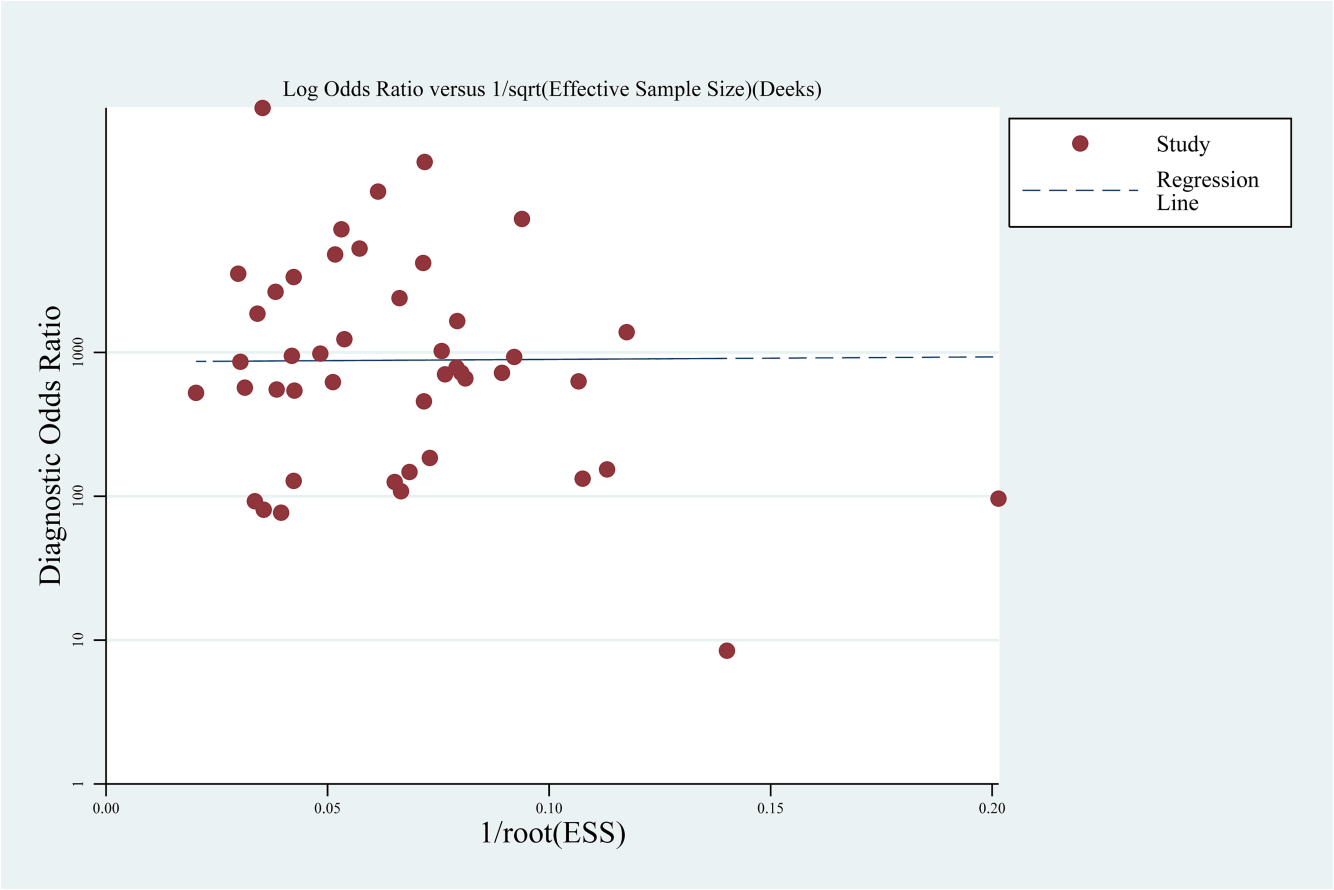
Deek’s funnel plot.

Deek’s funnel plot was used to assess publication bias, and the result indicated no significant publication bias (*p* = 0.973) (Figure 4).

## 4 Discussion

The rapid antigen tests for SARS-CoV-2 require no healthcare professionals to perform and have short turnaround time, and were therefore recommended by the governments in face of the overwhelming need for SARS-CoV-2 test during the COVID-19 pandemic (1). The accuracy of these tests compared to the “gold standard” were however unclear. Several previous studies have assessed the accuracy of rapid antigen tests, including a previous systemic review and meta-analysis by Xie et al. (12). However, Xie’s study included nasopharyngeal swab samples which have to be collected by healthcare professionals. In addition, a type of rapid antigen test included in Xie’s meta-analysis (LumiraDx SARS-CoV-2 Antigen Test) requires special equipment to read out results. Therefore, the results reported in Xie’s meta-analysis did not represent the performance of fully-self-performed rapid antigen tests. In this systematic review and meta-analysis, we focused on the performance of self-tested SARS-CoV-2 rapid antigen tests using nasal swab samples which allow fully self-testing of COVID-19.

After database searching, we identified 45 eligible studies. After pooling the performance results from 50,897 suspected COVID-19 patients, the SARS-CoV-2 rapid antigen tests showed overall moderate sensitivity (0.77) and high specificity (1.00), DOR (625.95) and AUC of SROC curve (0.9746). Xie’s meta-analysis reported similar sensitivity for nasal samples (0.79). In the subgroup analysis, samples collected by the suspected patients themselves showed higher sensitivity (0.80) and DOR (735.02) compared to samples collected by healthcare professionals (0.73 and 596.90, respectively). These results indicate that rapid antigen tests are reliable when samples are self-collected by the suspected patients, which further support the usefulness of rapid antigen tests during disease pandemics. In the three rapid antigen tests which have been intensively assessed (BinaxNOW, Panbio, and STANDARD Q), Panbio showed the highest sensitivity (0.83) and BinaxNOW showed the lowest sensitivity (0.69). Similar observations were shown in Xie’s study (12), except that all the pooled sensitivity was slightly lower (0.65, 0.73, and 0.70 for BinaxNOW, Panbio, and STANDARD Q, respectively).

In Xie’s study, the subgroup analysis involved different Ct cutoff values (<20, 20–25, 25–30, and >30). In our study, however, almost all the Ct cutoff values reported in the eligible studies were > 30. Therefore, we performed subgroup analysis on each specific Ct cutoff value (e.g., 37 and 40 as shown in Table 4). Similar as Xie’s study, higher Ct cutoff value (40) showed lower sensitivity (0.72), compared to cutoff value of 37 (sensitivity: 0.78). Compared to our study, in Xie’s study, the sensitivity of rapid antigen tests was much lower (0.24) when Ct cutoff values of >30 were used (12). For samples collected less than 7 days after symptoms onset, the subgroup analysis results showed high pooled sensitivity (0.93), which was higher than the reported sensitivity in the corresponding subgroup (≤ 7 days after symptom onset) in Xie’s study.

Region of study, percentage of patients with symptoms, and sample types for RT-PCR were not included in the subgroup analysis in Xie’s study (12). In our study, the subgroup analysis showed that compared to other regions (America and Asia), studies from Europe showed both higher sensitivity (0.83) and DOR (1041.31). Studies with higher percentage (≥ 50%) of patients with symptoms had higher pooled sensitivity (0.79) than studies with lower percentage (< 50%) of patients with symptoms, indicating better accuracy of rapid antigen tests in patients with symptoms.

Nasopharyngeal or oropharyngeal swabs are commonly used for the testing of SARS-CoV-2 using RT-PCR. In several studies assessing the accuracy of rapid antigen tests, however, nasal swabs were used for RT-PCR (3, 5, 11, 16–19, 21, 22, 24, 25, 31, 44–46, 52). A previous study showed that when self-collected nasal swabs were used, the sensitivity and specificity of SARS-CoV-2 testing using RT-PCR were 90.32 and 100%, respectively, compared to nasopharyngeal swabs (53), indicating existence of false negative when nasal swabs were used. In our study, the pooled sensitivity of rapid antigen tests was 0.80 when nasopharyngeal or oropharyngeal swabs were used for RT-PCR. When nasal swabs were used for RT-PCR, the sensitivity of rapid antigen tests dropped to 0.73, which might be caused by higher risk of false negative in RT-PCR results when nasal swabs were used. Since two separate nasal swab samples were usually collected for RT-PCR and rapid antigen test (3, 5, 11, 16, 17, 19, 22, 24, 25, 46, 52), failure in collecting COVID-19 virus in either of the two samples would lead to either false positivity or false negativity when RT-PCR is used as gold standard. The increase in the number of false negative samples may resulted in decreased sensitivity as observed in our results, while increase in the number of false positive samples did not change the specificity much due to the large number of true negative samples. These results indicate that nasopharyngeal or oropharyngeal swabs are preferable samples for RT-PCR while assessing the accuracy of rapid antigen tests.

In summary, results of this systemic review and meta-analysis showed overall high accuracy of self-performed SARS-CoV-2 rapid antigen tests. Compared to BinaxNOW and STANDARD Q, Abbott Panbio had the highest sensitivity and therefore more recommended. The results supported the collection of nasal swab by the suspected COVID-19 patients themselves. In addition, sensitivity of rapid antigen tests was higher in patients with symptoms. For the performing of “gold standard” RT-PCR, the standard sample type, nasopharyngeal or oropharyngeal swab, is recommended, and use of lower Ct cutoff value could help increase the sensitivity of rapid antigen tests. Samples collected within 7 days after symptoms onset showed high sensitivity (0.93). Limitation of this study may be the small number of studies in some subgroups of collection time after symptoms onset and cutoff for Ct value. In addition, many rapid antigen tests were only assessed in one or two studies, which cannot be individually analyzed in the subgroup analysis. More studies are required to further validate the results of this study and show more details in the subgroup analysis. This study only involved nasal swab sample results, while there are other sample types for COVID-19 rapid antigen test, e.g., saliva, throat swab which were relatively less studied. In a previous systemic review and meta-analysis, saliva and throat swab samples showed lower sensitivity (68 and 69%, respectively) than nasal swab (83%) (54). More investigations are required to further clarify the performance of rapid antigen tests using these samples types.

## Data Availability

The original contributions presented in the study are included in the article/Supplementary material, further inquiries can be directed to the corresponding authors.
